# Scientists benefit greatly from K-12 partnerships: the Panama Research Experiences for Teachers project

**DOI:** 10.1186/s12052-022-00177-z

**Published:** 2022-12-19

**Authors:** Bruce J. MacFadden, Claudia Vargas Vergara, Bradford T. Davey

**Affiliations:** 1 Thompson Earth Systems Institute (TESI), Florida Museum of Natural History, University of Florida, Gainesville, FL 32611 USA; 2 Office of Advancement, Santa Fe College, Gainesville, FL 32606 USA; 3 Technology for Learning Consortium Inc, Stuart, FL 34996 USA

**Keywords:** Biology, Earth sciences, Evolution, Geology, GABI (Great American Biotic Interchange), Paleontology, Professional development, RET (Research Experiences for Teachers), Scientist, STEM

## Abstract

Scientist-teacher partnerships are highly beneficial to K-12 STEM education. While much is known about the benefits for teachers in these partnerships, the corresponding benefits for scientists are less well known. With emphasis on the scientists’ perspective, here we describe our NSF RET (Research Experiences for Teachers) project consisting of five successive cohorts from 2012 to 2016. Coincident with a “once-in-a-century” expansion of the Panama Canal, the science research focused on the paleontology, evolutionary biology, and geology of this region to better understand the ancient Neotropical biota related to the Great American Biotic Interchange (GABI). In the field, scientists and teachers worked together collecting fossils and geological samples. Back in the K-12 classrooms, lesson plans related to their experiences were implemented and the teachers hosted scientist role-model visits. More than 30 scientists and 44 teachers participated in this Panama “GABI RET” project. Using a new validated survey developed during this project and focus groups, we explored the impact of this project, and in particular the perceived benefits accrued by the scientists. Our study confirmed that scientists felt they improved their communication skills, had a better appreciation for the K-12 teaching professions, greatly enjoyed working with the teachers, considered them colleagues, and many wanted to continue K-12 outreach as part of their careers. Overall, scientists perceived that they greatly benefited from these partnerships. In addition to describing their activities, they had numerous recommendations for similar partnerships in the future. For example, these include: (1) having more teachers participate in multiple cohorts, (2) continued opportunities for teachers to be involved in professional meetings, (3) ongoing webinars and face-to-face engagement, and (4) more diversity of racial and ethnic backgrounds, subjects taught, and regions represented. Although this case study was focused on the GABI RET, our results also potentially inform other projects that involve scientists’ education and outreach activities.

**Supplementary Information:**

The online version contains supplementary material available at 10.1186/s12052-022-00177-z.

## Introduction

Partnerships between scientists and K-12 STEM teachers are vitally important to enriching student learning in the classroom and supporting teachers in their instructional activities (Johnson 2017). Many programs have been developed over the years to promote these kinds of collaborations. The overall benefits of these programs and positive impacts on both the teachers and their students are widely recognized in the literature (NRC 2012; Gardner et al. 2022). On the other hand, little is known about the corresponding impact on the participating scientists. In this case study we demonstrate that they perceive great benefit from these K-12 partnerships.

Several recent programs have encouraged the development of scientist-teacher partnerships. A prime example of an effective strategic scientist-teacher partnership model is NSF’s GK-12 program (NSF 2021). Between 1999 and 2011, when the GK-12 program ended (Mervis 2011), more than 300 of these grants were awarded mostly to universities. In total, thousands of teachers and scientists were involved in GK-12 programs that ultimately impacted tens of thousands of K-12 students (Gamse et al. 2010; Ufnar et al. 2012). An important, overriding outcome of these GK-12 programs was a sense that scientists improved their communication skills (Cormas and Barufaldi 2011; Johnson 2017); this is a common characteristic of these and other similar programs, as also confirmed in this study.

Of more direct relevance here, NSF’s Research Experiences for Teachers (RET) program, which is currently active (e.g., NSF 2022), is another example of scientist-teacher partnerships in research collaborations and K-12 education. Similar to its companion program, Research Experiences for Undergraduates (REU), teacher participants in RETs are expected to learn how to do science through direct engagement with scientists and their authentic research. Active participation in the research enterprise is of great importance to teachers because in addition to teaching STEM content, they are also expected to deliver lessons plans that demonstrate the nature (or process) of science. Teachers can thus benefit from these experiences to increase their pedagogical content knowledge, which represents an integration of science content, pedagogy, students’ backgrounds and conceptions, and the specific learning environment (Shulman 1986, 1987; Cochran 1997). To the point of this paper, the corresponding benefits accrued to the participating scientists is less well known.

This case study therefore aims to describe, primarily from the scientist’s perspective, the development, activities, and outcomes of a five-year-long scientist-teacher partnership called the GABI (Great American Biotic Interchange) RET that included five cohorts between 2012 and 2016. Tanner (2000, p. 26) notes that: *Often descriptions of teacher-scientist partnerships have focused on the benefits to teachers and even more so to students*. Following this observation, our rationale is that in these kinds of partnerships, and teacher professional development in general (e.g., Tanner 2000; Tanner et al. 2003; Dresner and Starvel 2004), the emphasis for evaluation of outcomes is primarily focused on benefits for the teachers and their students, and less on the benefits accrued back to the scientists. However, as the GABI RET developed, we anecdotally observed immense satisfaction and engagement from the scientist participants. We, therefore, sought to further inform our understanding of the scientists’ benefits through this study.

Given the context above, our focus is on the impact that the GABI RET had on the scientists. To this end, the questions and outcomes that we investigated during this retrospective case study (Davey 2017) include:

1. In what ways did working with the teachers on their research project affect the scientists?
2. In what ways did the research experience affect scientists’ practice?

**Fig. 1:**
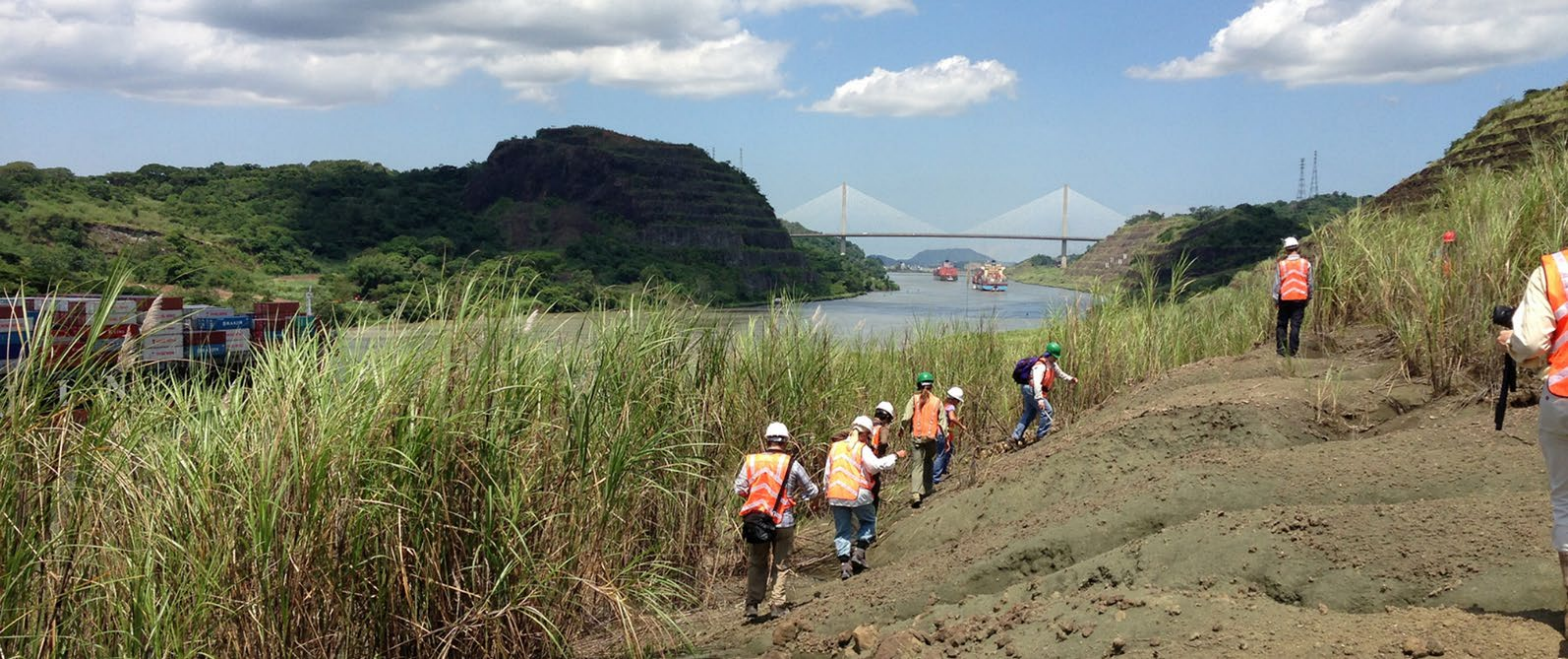
Scientists and teachers collecting fossils along the Panama Canal, 2013 (Joe Kays photo, University of Florida)

This program was initially the vision of Gary Bloom, Superintendent of the Santa Cruz, California, City Schools (now retired). He had an avocational interest in fossils, and during a trip to Panama in 2011, he volunteered in a paleontology laboratory at the Smithsonian Tropical Research Institute. His experience at that time was so positive that he wanted his STEM teachers also to have similar experiences participating in authentic research. He contacted the senior author, and together we developed cohorts 1 and 2 (Fig. 1) as supplements to the ongoing NSF-funded Panama Canal Project (PCP 2022); these were followed by an NSF GABI RET award that funded cohorts 3 to 5.

## STEM content: the GABI (Great American Biotic Interchange) and Panama connection

**Fig. 2:**
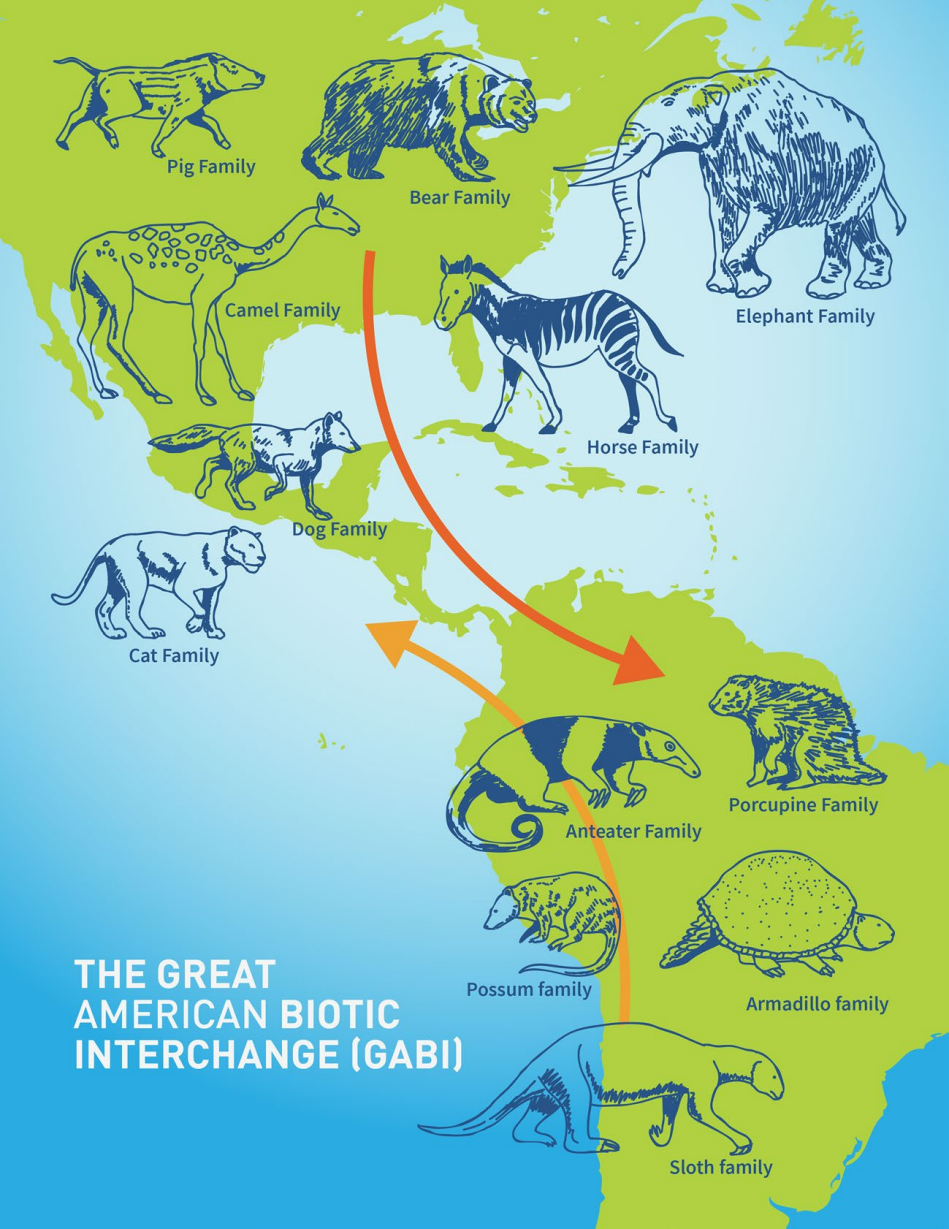
Graphic depicting the interchange of terrestrial mammals between the Americas as a result of GABI. Reproduced courtesy of the Smithsonian

The STEM content of this RET program was focused on the GABI (Fig. 2). Based on the fossil record and associated geological evidence, the Isthmus of Panama formed between about 5 to 3 million years ago (mya), resulting in a dryland bridge across Central America. This also facilitated active overland faunal dispersal of the native biota in South America to North America and vice-versa. For example, the presence of giant sloths and armadillos in North America during the Pliocene and Pleistocene epochs resulted from south to north dispersal. Conversely, the presence of horses, camels (llamas), proboscideans (elephants and their relatives), and several carnivoran groups in South America resulted from north to south dispersal of native North American faunas as they traversed the Panamanian land bridge (e.g., Stehli and Webb 1985; Webb 2006). Equally as fundamental to Neotropical biodiversity and evolution, the formation of the Isthmus of Panama closed the Central America Seaway during the Pliocene, between about 5 to 3 mya (e.g., Flynn et al. 2005; O’Dea et al. 2016). This final closure effectively severed marine biotic dispersals between the Atlantic Ocean and the Pacific Ocean (O’Dea et al. 2016).

The sequence of sediments exposed along the Panama Canal is primarily Neogene in age. Of relevance to the focus of our study, these range in age from about 24 to 18 million-year-old Miocene sediments exposed along the southern reaches of the Canal, including the presence of early Miocene land mammals with North American affinities (Stewart et al. 1980; Whitmore and Stewart 1965). Along and adjacent to the northern reaches of the Panama Canal, 10 million-year-old late Miocene sediments have produced highly diverse invertebrate and shark faunas from the Gatun Fauna (Woodring 1957; Pimiento et al. 2013). From 2008 until 2014 the Republic of Panama undertook a major expansion of the Panama Canal, and in so doing, uncovered new and important exposures of these fossil-bearing sediments. Seeing this “once-in-a-century” opportunity, NSF funded a project to investigate these fossils and their importance for education and international partnerships with the Panama Canal Project (PCP 2022). This initiative led to the student–teacher partnership that developed into this Panama GABI RET.

Within this context above, the RET participants had the opportunity to learn about the GABI, which is widely considered by scientists as one of the classic “natural experiments” in the history of life on earth (e.g., Stehli and Webb 1985; Webb 2006). The STEM content of the GABI spans a broad range of the natural sciences, including evolutionary biology and earth sciences (geology). Participants also learned about, and were exposed to, all aspects of the process (nature) of science, ranging from field explorations (Fig. 1), to systematic identifications, to the scientific interpretation of the importance of these fossils. Teachers applied this learning to lesson plans, many of which are freely available on-line (FLMNH 2022), whereas the scientists included these experiences in their research and related activities. Of particular note, many of the scientists participated in K-12 classroom visits during the GABI RET (also see further description of these activities below).

## RET: conceptual framework

“Scientists and engineers working in partnership with local teachers represent an essential new force that will be required for effective science education reform.” (Alberts, 1994, n.p.)

### Scientist-teacher partnerships

As emphasized by former National Academy of Sciences President Bruce Alberts above, effective scientist-teacher partnerships can potentially have a positive effect on STEM instruction. Therefore, this conceptual framework has been known for decades and funded by many programs, frequently at the federal level (e.g., National Science Foundation) and numerous philanthropies, e.g., HHMI (Howard Hughes Medical Institute).

**Fig. 3:**
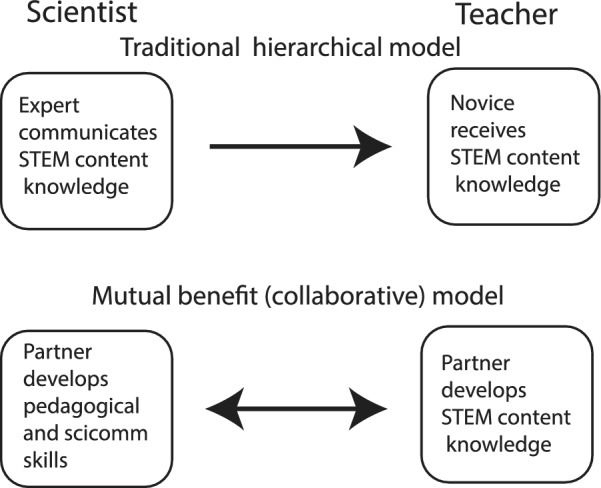
Comparison of the traditional hierarchical vs. the mutual benefit model of scientist-teacher partnerships

Although the primary focus of this paper is the effect that the RET had on the scientists, some context from the teachers’ side of this partnership is germane. Scientist-teacher partnerships have essential components that relate to what is known about successful teacher professional development (PD) and the concept of mutual benefit and knowledge reciprocity. For these partnerships to be successful, there are specific essential criteria. First and foremost, successful teacher PD should not be considered a “one-off,” but rather an ongoing process of partners and collaboration (OECD 2009). Likewise, the classic concept of scientist-teacher PD representing primarily an expert-novice learner model with a unidirectional flow of information and expertise is outmoded and has given way to non-hierarchical collaboration (Fig. 3; MacFadden 2019; Abramowitz et al. 2021). Johnson (2017) rightly refers to this process as a community of practice (e.g., Wenger et al. 2002), a well-known concept in learning communities and one in which there is a shared purpose and active interest in a particular topic among all stakeholders. Another important concept is the notion of balance (Van Schaik 2017), i.e., optimally, each partner contributes and benefits from the partnership to result in meaning to them. If all of the benefits accrue to some, but not all partners, then such an imbalanced collaboration is not as strong as it could be, and there is less probability of it being sustained.

### Mutual benefit

In any partnership, one must consider what all of the participants and stakeholders are getting from it (MacFadden 2019). In the traditional the expert/novice model, the primary benefit of scientist-led teacher PD has been directed to the teachers. These benefits of teacher-focused PD are significant, including increased confidence to teach science, increased STEM identity, connection with scientists and other energized teachers (e.g., Dresner and Starvel 2004; Johnson 2017), and overall increased pedagogical content knowledge (Shulman 1986, 1987).

On the other hand, while studies describing the outcomes for scientist participants are certainly less well-known, an emerging body of literature discusses these benefits. For example, scientist benefits include better communication skills, a better appreciation of the teaching profession, and an altruistic sense of giving back to society via K-12 teacher and classroom activities (Gamse et al. 2010; Cormas and Barufaldi 2011). In one of the first studies that formally evaluated outcomes of the benefits, Tanner (2000, p. 25) states that “scientists benefit enormously from these partnerships, as scientific professionals, as future teachers at the undergraduate level, and as individuals.”

### Best practices

Of relevance here, and as will be further developed below, from studies of scientist-teacher partnerships, recurrent themes and tips are highlighted on how to develop successful programs and potentially optimize outcomes. These include: (1) a non-hierarchical learning model in which all participants are valued for their knowledge and contributions to a particular project. (2) Partnerships that are not “one-off,” but instead viewed as an ongoing collaboration with sequential activities. (3) Partnerships organized around the concept of cultivating a sense of community (Johnson 2017; Warwick et al. 2020). We will also see below that these best practices, generally developed from prior studies, also resonated from the scientists’ point of view during the GABI RET.

## Project components and activities, with emphasis on the scientists

The structure of the GABI RET program activities included the development of five separate year-long scientist-teacher cohorts. The cohort was initiated with the authentic field research experience during the summer of each year (2012 to 2016) in which the scientists and participants traveled to Panama for between 10 to 14 days. In the field they collected fossil vertebrates, invertebrates, and plants and associated geological samples primarily around the Panama Canal. When they were not conducting field work they attended talks, seminars, and laboratory tours at the Smithsonian Tropical Research Institute (STRI), which conducts research on Neotropical natural science and archaeology. While in Panama, most evenings, the scientists and teachers assembled back at the hotel for “poolside chats” to reflect on what they learned that day and to plan for future activities.

The scientists worked alongside the teachers and mentored them, mostly via sharing their expertise and to help in the identification of fossils that were being collected. The scientists held impromptu chats with the other scientists and teachers on the outcrops to explain about their own research. Back at STRI, some of the scientists also presented their research at the weekly “Paleotalks,” which consisted of semi-formal 45-min-long talks to an in-person audience consisting primarily of our cohort teams (both scientists and teachers), but oftentimes also including other members of the STRI community. The cohorts also attended the weekly institution-wide STRI general seminar series, typically on a broader range of topics related to Neotropical biodiversity, ecology, and evolution, and these were followed by a popular social hour afterwards.

The intensive field immersion experience in Panama facilitated the scientists and teachers to form lasting professional connections of mutual benefit. Thus, after the summer experience in Panama, many of the scientists and teachers maintained communication during the academic year, mostly via email. Some of the scientists were invited to visit the classrooms, both for their content expertise and as role-models. This component evolved organically in such a way that the scientists, i.e., mostly graduate students, interns, and other early-career professionals working on the Panama project, were invited by the teachers into the classrooms as near-peer role models. Thus, in addition to delivering science content, they also discussed their STEM career journey, science identity, and 21st-century careers. These visits were not an a priori planned component of the project, but as was revealed in the evaluation results (described further below), they were mutually beneficial and highly valued.

## Participant recruitment and characteristics

Although the primary focus of this case study is the scientists’ point of view, this section first briefly reviews the recruitment and related characteristics of the GABI RET teacher participants to provide some relevant context for a more in-depth discussion of the scientists that follows. In discussions about scientist-teacher partnerships, these two groups, i.e., the scientists on the one hand and the teachers on the other hand, might be thought of as homogeneous groups, however, in certain aspects, they may be heterogeneous. Individual participants, may, for example, have different STEM content and focus, range from early to late career stage, and also potentially come from different backgrounds and experiences. It is therefore important to keep this context in mind when discussing these group attributes.

### Teachers and K-12 educators

Teachers for cohorts 1 (2012) and 2 (2013) were recruited by invitation from Gary Bloom, then Superintendent of the Santa Cruz (California) City School District from his district and the adjacent Pajaro Valley Unified School District (PVUSD). As the GABI RET program developed in subsequent years (3 to 5; 2014 to 2016), teacher recruitment included recommendations from participants, “word-of-mouth,” and a more formal application recruitment process, the latter of which was reviewed by the scientists and then the selections were made. Teachers came from public elementary, middle, and high schools and primarily taught biology, earth science, and geology, and environmental science. However, some other disciplines were also represented (chemistry, math, physics, and art). They also represent different career stages. As presented in Table 1, 47 teacher participants included 44 unique teachers, with 3 teachers participating in a 2nd cohort as a “teacher-leader.” All teachers successfully completed the entire six-month-long professional development cohort cycle (July through December).

**Table 1:** Number of participants and total by cohort and category in the 5-year-long GABI RET project

Cohort (Year)	Teachers^a^	Scientists^b^	Other participants^c^	Total
1 (2012)	5	5	2	11
2 (2013)	8	4	3	15
3 (2014)	10	3	1	14
4 (2015)	11	12	2	25
5 (2016)	13	12	1	26
Total	47	36	(9)	83 (92)

^a^Teachers included two administrators, i.e., 1 superintendent (2012) and 1 assistant superintendent (2013)

^b^Scientists included professors, other scientists, postdoctoral fellows, undergraduate students, graduate students, and interns in Panama. The exact number in this category was fluid during each cohort and therefore difficult to accurately determine because some did not participate in the entire cohort experience; the numbers reported above are likely a minimum

^c^Other participants included spouse and photographer (2012), journalists (2013), citizen scientist (2015), and project manager (2012 to 2016)

### Scientists

Scientists are defined as any GABI RET participant with science content expertise related to their research, including professors, other scientists, postdoctoral fellows, undergraduate students, graduate students, and interns in Panama. Like the teachers, these scientists are heterogenous in their career stage and demographics, but are united as a learning community of practice (e.g., Wenger et al. 2002) with a shared interest in the domain of paleontology and related disciplines. These 36 scientist participants (Table 1) included about 30 unique scientists, with some scientists participating in multiple (up to 5, in the case of the senior author) cohorts. The exact number of scientists is difficult to accurately determine because, although some scientists participated during the entire cohort cycle, others came and went, e.g., participating in only the field aspect, lab activities at STRI, perhaps subsequent school visits, or the final wrap-up session at the end of a cohort.

Scientists were recruited based on their relevant expertise and interest in participating on the GABI RET project. They were selected by invitation from the GABI RET team leaders based on how the scientists were perceived to be able to contribute to the scientist-teacher experience. Some scientists were already in residence, i.e., working at STRI, whereas others travelled with the cohort team to Panama. With regard to scientist demographics, 56% (20) were early career (i.e., undergraduate students, interns, graduate students, or postdoctoral fellow) scientists, most of whom were training to become professional paleontologists or geologists. A total of 39% (14) were women and 28% (10) were Hispanic or Latine.

### Other participants

Other participants, i.e., neither fulfilling the role of teacher nor scientist, participated primarily on the Panama field trip, but not during the remainder of the six-month-long PD process. This included a spouse and photographer in 2012, two journalists in 2013 (editor and photographer, see Kays 2014), and a citizen scientist in 2015 who had collected fossils in Panama in the 1950s as a young man living in the Canal Zone. A project manager (author Vargas-Vergara [Grant]) participated from 2013 to 2016 and remained engaged throughout each cohorts’ activities. She later went on to receive her PhD in educational technology, largely influenced by her participation in the GABI RET (Grant 2020).

## Project evaluation

Two phases of evaluation were conducted during this project: formative and summative. Formative evaluations were primarily done with e-surveys developed with the intention to iteratively improve upon, or learn from, the individual experiences of cohort participants. These were conducted after specific project events and, in particular, after the cohort traveled to Panama and near the end of the six-month-long cohort PD cycle. We also received informal, qualitative feedback from teachers after other activities, such as the scientist role-model visits to schools. The results of these formative evaluations were documented in the NSF annual reports.

The summative evaluation was conducted by TLC (Technology for Learning Consortium, Inc), of which author Davey is a Principal. In addition to providing evidence of the project activities for NSF reporting, this evaluation was intended to inform our retrospective case study represented here. Data were primarily collected from e-surveys and focus groups, the latter of which were administered during a project wrap-up at Ghost Ranch, New Mexico, in July 2017. The analysis included a mixed-methods approach (Frechtling 2010) to collect quantitative and qualitative data from both the scientists and teachers. Quantitative data included responses to questions that required Likert-like comparisons (Croasmum and Ostrom 2011). Qualitative responses were taken from the open-ended survey questions and focus group transcripts and then analyzed using discourse analysis to identify themes and patterns among the respondents. Davey (2017) describes the GABI RET summative evaluation process and findings in far greater detail than is intended here. All evaluations done during the GABI RET were approved by the UF Institutional Review Board (UF IRB-02 project 2014-U-0267).

With regard to the primary focus of this paper, our survey of the learning research literature did not find a validated survey that would be appropriate to evaluate the scientist group. Therefore, we developed one during this research project (see Additional file 1). Our survey development included face validity, content validity, construct validity, test–retest reliability, and internal consistency reliability (Burton and Mazerole 2011). Furthermore, this process had the following steps: (1) defining the constructs to be examined, (2) generating items to test these constructs—Delphi, (3) pilot testing, (4) analysis of pilot data, and (5) retest-test data collection and analysis. The mean test–retest reliability for the quantitative survey items was 0.93, indicating a high correlation. Davey (2017) provides a detailed description and results of this survey validation process but in short, the survey had a total of six factors (professional, mentoring, teaching, personal, participation with K-12, and understanding of K-12) with between three and six items per factor with a final total of 11 questions with 31 items. The instrument was validated internally with 17 participants over two iterations approximately five months apart. Table 2 below lists the different aspects of the instrument development, participants, actions, and results.

**Table 2:** Instrument development and validation

Study Design	Participants	Actions	Results
Development	Researcher	Review of literature, defining constructs, item development	Draft scientist survey
Delphi	Six professionals	Three iterations of suggestions and edits shared virtually through e-mail	Final scientist survey
Pilot testing—round 1	17 Scientists	Scientists took survey	Summarized findings
Pilot testing—round 2	17 Scientists	Scientist took the survey a second time—matched pair analysis performed on results from round 1 and 2	Mean test–retest reliability for the quantitative survey items = 0.93
Professional review	Six professionals	Shared findings with professionals from Delphi study	Shared confidence in findings and instrument

## Results: scientists’ perspective

The primary aim of this report is to better understand the impact and benefit that the GABI RET had on the scientists. This task was addressed via mixed methods, i.e., quantitative and qualitative surveys (N = 15) and focus group discussions (N = 9) that were administered, respectively, via e-surveys and during the project-closing Ghost Ranch summit. The N = 15 represented all of scientists that responded to the e-survey; this sample represents the great majority of those actively engaged near the end of the project. The N = 9 sample for the focus group discussions represent all the scientists that participated in the summit.

The validated survey (Additional file 1) asked scientists to identify how they were affected (STEM identity, understanding of the nature of science, practicing science) and what experiences were related to those effects (e.g., international immersion, working with teachers, sharing science and practice). In the focus groups, scientists were asked about how the effects identified translated into changes in their practice and how they now view the importance of exposing teachers to science, the nature of science, how they practice science, and their role as scientists. To this end, during this retrospective case study, we investigated (also see Introduction above) the following questions:

## Question 1: In what ways did working with the teachers on their research project affect the scientists?

### Sub-question 1a. Did your understanding of teaching and learning change as a result of working with your teacher partners? If so, how?

Based on the focus group discussion, all scientists reported that working with the teachers changed their understanding of teaching and learning, particularly in K-12 settings. The scientists spoke about learning how to develop lesson plans and incorporate standards (e.g., NGSS 2013), making opportunities for learning fun and engaging, and emphasizing the importance of classroom management. The scientists also enjoyed the excitement the teachers brought with them into the field. A selection of their verbatim, open-ended responses is presented in Table 3. In this and subsequent tables, responses were selected because they appeared to be the most informative answers to the questions. In some other cases, the answers were considered duplicative and therefore interpreted not to be necessary to repeat. These answers therefore represent a subset of all of the responses, the latter of which can be retrieved from Davey (2017)

**Table 3:** Selected scientists’ responses to sub-question 1(a)

• Yes. I learned how lesson plans were created, the standards they had to adhere to and how to explain a complicated subject to someone with no or very little prior knowledge on the topic and make it fun, engaging and learn from it
• Yes, I became more aware of the importance of classroom management and teaching to learning standards, such as NGSS and Florida's Sunshine State Standards
• Yes. As a scientist, I did not have much experience in understanding how/what K12 educators need for meaningful collaboration
• GABI-RET is pioneering teaching experiences that allow not only K12 teachers but also scientists, like myself, to become better learners and communicators through collaboration thus producing more effective
• Absolutely- I am now much more aware of the opportunities and limitations associated with teaching at the K-12 level, as well as the power of engagement through interactions with the scientific community and hands on field based learning
• Definitely, I am primarily a museum curator/ researcher and do very little teaching at any level, but mostly college not K-12. It was great working in the field with the middle and high school science teachers and experiencing their excitement to learn new things. I suppose the most important thing I learned was how important field and hands-on experiences are to teaching

### Sub-question 1b. If you had an opportunity to visit a classroom, how did your visit impact you? If you visited more than once, how did your visits change over time?

Eleven of the 15 scientists survey respondents (73%) had an opportunity to visit a classroom, either in-person or virtually. Most of them visited more than one classroom or one class multiple times, with one scientist visiting nearly all of the teachers’ classes at some point. The scientists were generally surprised by the level of engagement and excitement of the students and the types of questions they asked. From these teaching experiences, scientists began to think about teaching in both formal and informal settings, changed their understanding of how to work well with students, how to best communicate with them and convey their ideas and findings, and the impact of the GABI RET experience on the teachers and their classes.

**Fig. 4:**
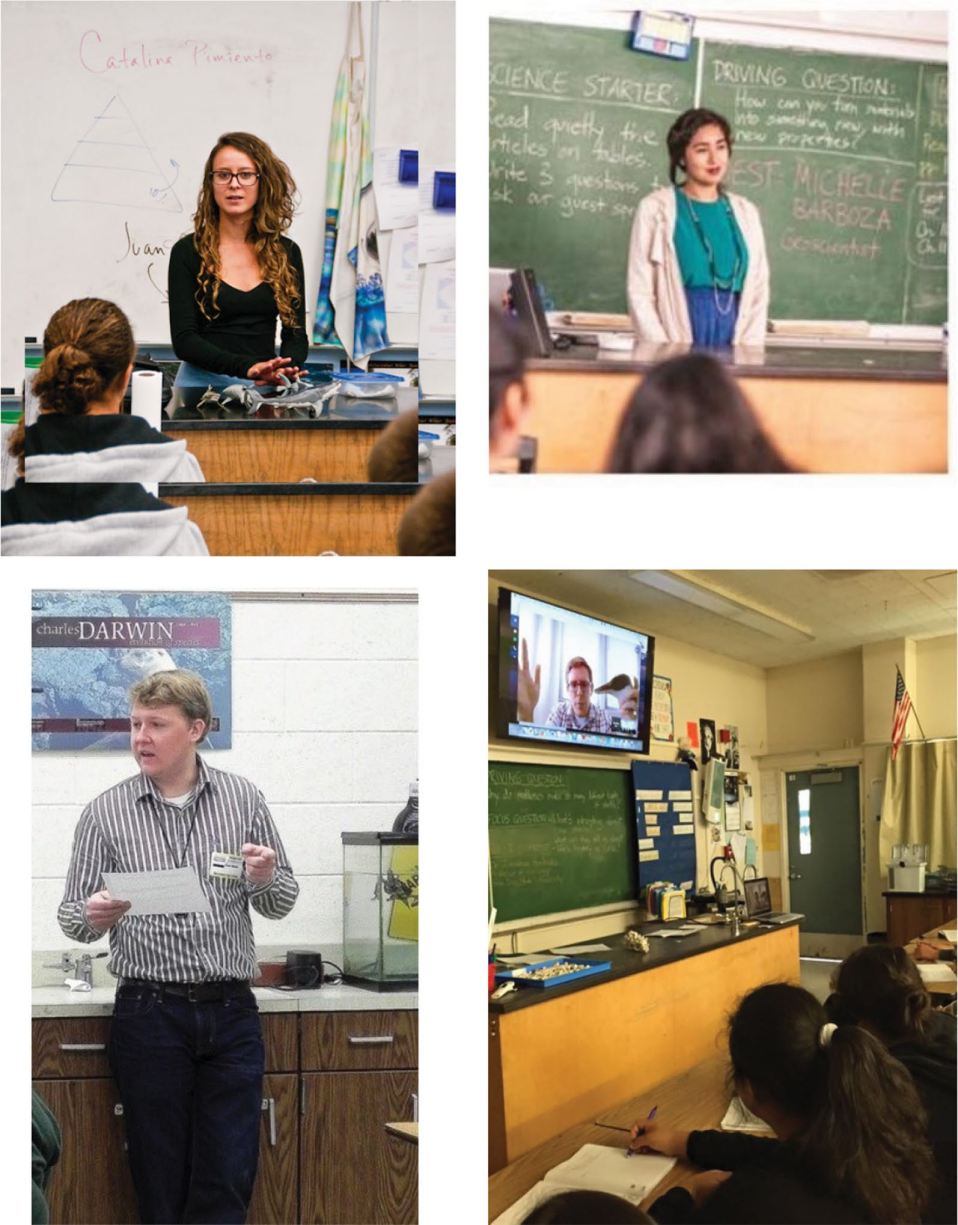
Examples of scientist role-model visits to California schools, including in-person and virtual

Consistent with best practices for professional development, many scientists continued their association with specific teachers. The 15 scientists who responded to the e-survey reported a total of 177 continuing collaborations (mean = 10) with their teachers, and some collaborations continued past a particular cohort year into successive years. These collaborations most frequently included continuing input on lesson plans, role-model classroom visits (Fig. 4), and presentations at professional meetings.

During the focus group, we further explored the overall benefits that the scientists had from working with the teachers. All nine of the focus group scientists (100%) reported visiting a classroom or having the students come to them. Many of the scientists reported being interested in teaching and working with students, with this interest being increased or activated by participation provided the GABI RET. Notably, the scientists reported gaining valuable experience with presentation and communication skills.

The role-model visits developed organically after the scientist-teacher cohorts bonded. The scientists also talked about being role models for the students, considering their work in a broader context, and learning to work with and engage students in and out of the classroom. To quote one scientist in this context: *“All of us have some level of interest in teaching, but don’t often get any training on how to teach. Working with the teachers and visiting their classrooms has taught me more about teaching than I ever got as a TA* [Teaching Assistant].”

The scientists who visited a classroom more than once reported that they became more comfortable working with the students and were able to better communicate their ideas and answer the students’ questions. For one scientist (a graduate student), the numerous classroom visits changed their intended career direction from science-research focused to K-12 education. Selected verbatim responses are presented in Table 4.

**Table 4:** Selected scientists’ responses to sub-question 1(b)

• I did a Skype visit with a 3rd grade classroom. I was surprised by the level of questions being asked. The questions were much more sophisticated than I expected from 3rd graders. I realized that I had misconceptions about what elementary school students already knew about science, ecology and animal behavior. I did not get a chance to visit with the same class again
• Interacting with students in the classroom helped me realize that I want K12 teaching to be a large component of my career. However, after multiple classroom visits I felt that I would prefer teaching in an informal setting, such as a museum
• I really enjoyed sharing my knowledge and perspective regarding my journey as a paleontologist. It was great to see students excited about science!
• The visits were amazing! Over the course of multiple visits, I got to understand my audience better and tailored my speeches to be about what they wanted to hear, not just what I thought they wanted to hear
• I visited teachers’ classrooms through video conferencing; it was a wonderful experience to share my research with students, as well as to answer their questions about my research
• It impacted me deeply, but hard to put it in words. It was just a very rewarding experience as I could see excitement on students and real-time change of perceptions
• I never visited a classroom but I did do two Skype presentations to a second grade science class. Having the opportunity to interact with the children and see their excitement for science was quite motivational. I really am living the dream!

## Question 2: In what ways did the research experience affect scientists’ practice?

### 2(a). Explain how working with teachers on the Panama project influenced your implementation of the scientific process in your own work.

The focus group revealed that working with the teachers during the GABI-RET profoundly impacted the scientists’ own practices. When asked about the influence working with teachers had on their work, the scientists wrote about ensuring their work was more clearly explained and accessible, changes in their “end goal” for their work, and considering the broader impact of their work. Scientists frequently described improvements in their ability to communicate their work. Communication is something that scientists are often challenged with, and strategic ways to help them become better communicators are generally welcome. The scientists also wrote about involving teachers more in their research, how to translate their scientific work into classroom activities, and changes to their scientific writing style. Selected verbatim responses are presented in Table 5.

**Table 5:** Selected scientists’ responses to sub-question 2(a)

• I no longer consider a publication in a peer-reviewed journal as the end goal for a research project. Now I see it as a classroom lesson. I strongly believe that any research project can be translated into a K12 lesson
• I think that working with teachers has helped me learn how to communicate science to people in a more clear and concise way. Talking with the teachers allowed me to see how most people view and interpret scientific principles, which is important to know when explaining science to the public
• I found myself considering how I might involve teachers in my own work in the future. I also now consider what types of tasks are easily transferable to a classroom setting where students could participate in data collection
• I now not only feel more comfortable sharing my own research with broader audiences of all ages, but also see the necessity of doing such. Achieving impactful 'broader impacts' initiatives now seems much more attainable and I am even more comfortable/confident training others of varying levels of experience in the methods and process associated with my work
• Teachers assisted with collection of the fossils that I have subsequently used in my research

### 2(b). What did you learn about mentoring from working with the teachers during this experience? What surprised you most about these interactions?

In the focus group, when asked about what they learned about mentoring from working with the teachers, the scientists shared how their perception of the relationship changed from mentor–mentee to mentor-mentor (Fig. 3). The scientists felt they learned as much from the teachers as teachers were learning from them. Scientists’ perceptions of mentoring thus changed from a one-directional relationship to a two-way relationship where both parties benefit (Fig. 3). The teachers learned skills and gained valuable field experience while the scientists observed the teachers' patience, passion and excitement, willingness to learn and grow, and the importance of having hands-on experiences. Selected verbatim responses are presented in Table 6.

**Table 6:** Selection of scientists’ responses to sub-question 2(b)

• My experience working with the teachers was more like a mentor-mentor relationship. I provided science content, but they provide teaching expertise. I've been surprised by how well connected many of the teachers have remained with FLMNH and their continued interest in developing new paleontology lessons
• I learned that most of the teachers were incredibly eager to learn about the way scientists gather data and interpret it. I had a great time explaining and demonstrating the research process
• …Without a doubt, the passion that I witnessed amongst the teachers in my group surprised me the most! This extremely prevalent passion just proves the value of GABI-RET. Five cohorts and there is still an army of STEM teachers longing to be part of GABI-RET
• I learned that when teachers are able to participate in science, the stories and pictures they bring back to the classroom can be just as engaging to the students as if they had gone on the trip themselves. I also saw how participating improves teachers understanding of the scientific process that they are tasked with teaching
• I learned that the teachers are highly motivated to learn as much as possible, and to pass along this new information to their students. With one notable exception (my high school biology teacher who got me interested in natural history), I don't remember my teachers in middle and high school being that interested or motivated
• The questions asked by the teachers about my research were excellent—these questions forced me to think critically about areas of my research that I did not understand well enough
• I learned how much teachers sacrifice for their students, and I was surprised to find this is true across teachers from all over the country

**Fig. 5:**
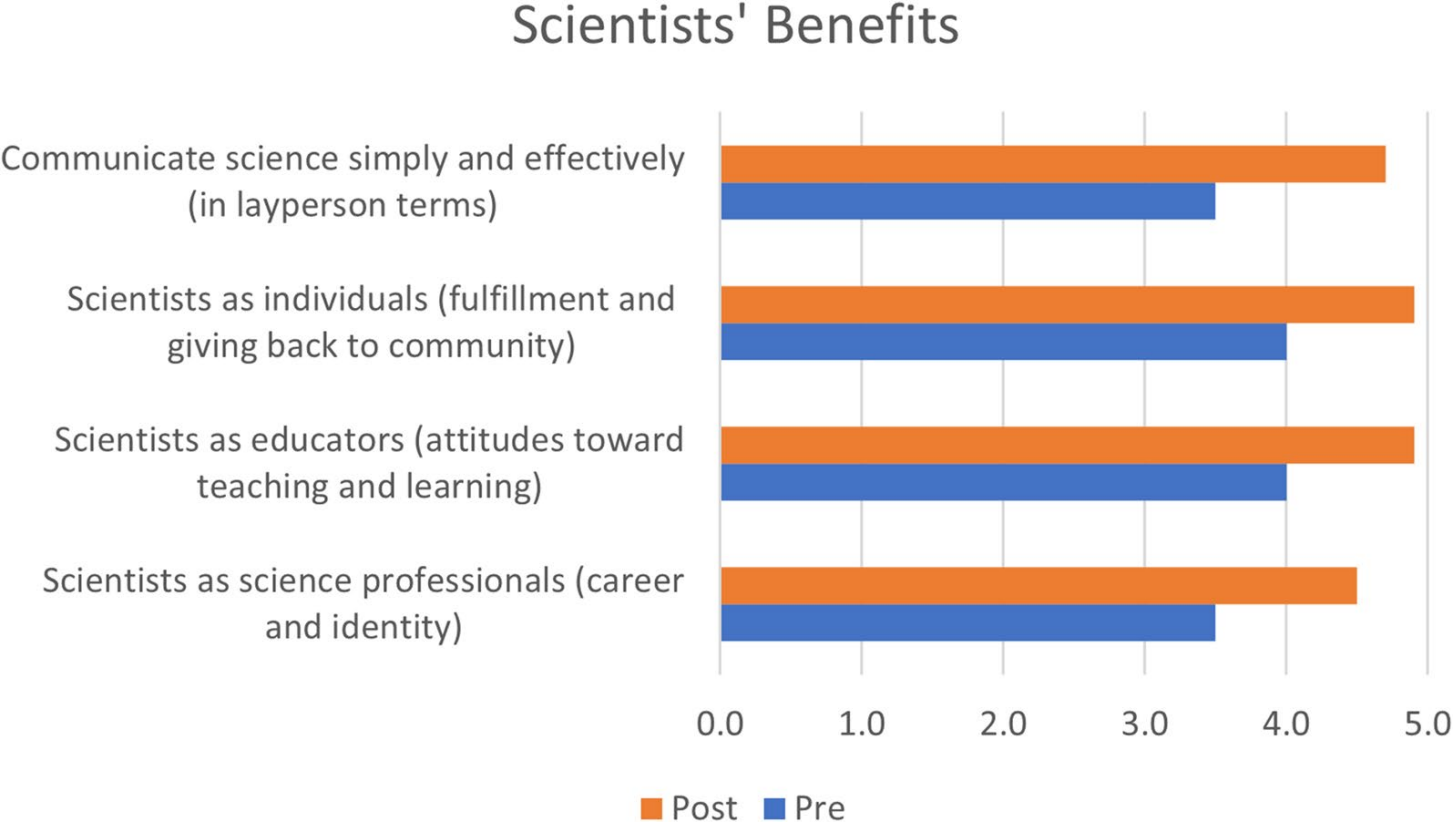
Gains in selected benefit attributes for the scientists (N = 15)

In addition to the data resulting from the validated survey and focus group described above, we also conducted a pre-and post-experience scoring using a Likert-like scale (5 = Strongly Agree, 4 = Agree, 3 = Neither Agree nor Disagree, 2 = Disagree, 1 = Strongly Disagree) with the 15 scientists. In one of these examples, our results indicate a statistically significant (p < 0.01) change from pre- to post (Fig. 5). This also demonstrates that the scientists mutually benefitted from their GABI RET experience.

## Discussion

### Recommendations: scientists’ point of view

During the focus group discussions at the Ghost Ranch summit, the scientists had a series of recommendations, some of which were already considered, and others that were new and relevant to program improvement (Table 7). We will address many of the enumerated recommendations below (1 and 2; 5 through 10), as follows:

**Table 7:** Scientists’ recommendations for future RETs

1. More diversity of educators (regionally and subjects taught)
2. Teachers from states where fossil digs occur
3. Develop a cohesive story for the program and experience
4. Focus on the science and engage the teachers in the discussion
5. Engage teachers in professional talks and experience
6. Find a way to allow teachers to take fossil samples back to their classroom
7. 25% returning teachers; have them present their experience to the new teachers
8. Consider pairs of teachers from the same school
9. Present webinars prior to the face-to-face for sharing information, covering preliminary topics, etc
10. Track collaborations and teach teachers how to prepare a professional poster and talk

Responses 1, 2, and 5–10 are discussed in the text (3–4) are not discussed because these are more general)

(1) With regard to diversity of the educators, in addition to different regions and subjects, we also would strive to bring more racial and ethnic diversity into our programs. The GABI RET teachers were overwhelmingly White (86%) and female (79%). With regard to ethnicity, 7% were Hispanic/Latino. From this, we realize that one goal for the future would be to make the demographics of the participants more closely reflect those of the region from which the teachers are recruited in order to better reflect alignment with student demographics.

(2) With regard to participants from the field area, when we were in Panama, we reached out to local schools. On a few occasions, we were able to visit schools to implement some lesson plans or have some of the students, teachers, and scientists work together on a field trip, as we did in the Gatun Formation in Panama. This was rewarding for the local community, many of whom did not know about the rich trove of fossils that existed locally and what they meant for the science of paleontology.

(5) Engage teachers in professional talks and experience. Typically, as these kinds of projects mature, the teachers are back in school focused on the lesson plans and the scientists are focused on their research. We did not build in the opportunity for teachers to present at professional meetings, but in a few instances we did fund travel for them to attend and co-present at meetings. We agree that this should be built into the project plan from the beginning.

(6) Allowing the teachers to bring back fossils into their classrooms. Most of the fossils were primarily collected under the auspices of a research permit. Nevertheless, to a limited degree, at some localities teachers were allowed to bring back representative fossils for instructional purposes. We also innovated during the GAB-RET by 3D scanning relevant fossils that could be printed out in classrooms (Ziegler et al. 2020), for use during role model visits to the teachers’ schools. Representative fossils have also been uploaded to Morphosource (2022), a digital repository for scanned images.

(7) Having repeat teachers in successive years is a good practice that we implemented each year during cohorts 2 to 5. This practice follows the concept of a teacher leader (Anonymous 2019), one who is already familiar with the program activities and goals. Thus, the value of this teacher-leader was the prior experience and mentoring brought to the next cohort. We identified one or two teachers from each cohort and invited them to return for successive year’s participation in the entire cycle. Although we did not have the 25% recommended by the respondent above, mainly because we wanted to maximize the total number of teachers involved, the teacher-leader model seemed to be effective for the GABI RET.

(8) Pairing teachers from the same school is an excellent idea, and we consciously did this, mostly by inviting teachers from these schools in successive cohorts. Several of these pairings were beneficial and we observed, for example, biology teachers at the same high school working together on GABI lesson plans and co-hosting scientist role-model visits to their schools. In retrospect, it may have been more effective if more teachers from the same school participated as members of the same cohort. In so doing, they could have formed a working partnership at the beginning of the cohort experience in Panama, rather than wait until they were both back at the start of the school year.

(9) The idea of hosting webinars and periodic check-ins is excellent. During later cohorts, we tried some monthly web-based check-ins, but these met with limited success. The challenge in this regard was that the participants were from different time zones, so finding a suitable time for everyone was nearly impossible. We thus made these an optional expectation of participation. In retrospect, we should have made these required as a program component. A decade ago, when we started the GABI RET, the use of online communication was less common than what we know today is now standard practice, particularly due to the impacts of COVID-19 (Hartshorne et al. 2022). We would implement expected and periodic web-based meetings to introduce the project and the scientist and teacher participants before the fieldwork and afterwards to keep the community of practice active for lesson plan development and implementation during the school year.

(10) We tracked collaborations and evaluated them (Davey 2017). We were, however, less intentional in encouraging teachers to present at professional meetings and such encouragement was done more on an ad hoc basis. We sent some teachers to professional meetings, but were likewise not intentional in helping them prepare unless they requested help from a scientist. In the future, being more deliberate would unquestionably become a best practice if teachers presenting at professional meetings is a project goal.

In total, these scientist recommendations all make sense and serve as further evidence of best practices for sustained and successful scientist-teacher partnerships in the future. In addition, a core best practice is to mutually respect both the teachers’ (Warwick et al. 2020) and scientists' time, both sets of which have many other demands. In so doing, the partnership will be mutually beneficial.

### Limitations and challenges

A major limitation of this retrospective study is that it was undertaken near the end the project (also see Ufnar et al. 2017). The reason for this is that we did not anticipate such a strong positive response from the scientists in terms of the great benefit that they derived from their participation in the GABI RET. We therefore had to use the data collected during the summative evaluation (Davey 2017). As such, we were not able to develop an a priori research design that would have allowed us to follow more rigorous analyses expected from educational research. While we have collected significant qualitative data from the open-ended responses during the summative evaluation, the relatively small sample size did not allow formal quantitative analysis.

Furthermore, our retrospective results did not allow for further investigation of different scientists’ perceptions, given the fact that they ranged from early to late career stages. The early career participants may have been more effective as near-peer role models in the classroom and more forward-thinking about how their individual experiences with science-teacher partnerships might be changed in the future. Later career respondents may have been more reflective of their experiences as contributions to benefit society and a sense of social responsibility. These aspects of the scientists’ perceptions would likely be of interest if in the future a follow-up study is done with a larger sample size that could compare differences within group participants.

## Concluding comments

Our GABI RET provides a clear example of how and why this partnership offers mutual benefit, and of particular relevance to the questions posed in this case study, the previously little-known significance of the impact on scientists. Our study further documents specific outcomes accrued to the scientists and reinforces other literature (e.g., Tanner 2000; Tanner et al. 2003) indicating these benefits. Not surprisingly, the GABI RET scientists felt they developed stronger communication skills, stronger STEM identity, and a better understanding of the practices and challenges of the K-12 teaching profession (e.g., Johnson 2017). In addition, the GABI RET also demonstrates innovation in these partnerships, i.e., the scientist role-model visits to the classrooms that were an unanticipated project component. In addition, best practices, such as a collaborative model, teacher leaders, and an ongoing (rather than one-off) process of sustained professional development can make these scientist-teacher partnerships more effective and successful for all participants.

Our study was opportunistic, i.e., we did not fully realize the value of the GABI RET to the scientists until near the end of the project. We therefore had to choose a retrospective design (Ufnar et al. 2017) for this case study, which we felt has value for the scientific community. In the future, other projects involving scientist-teacher partnerships that seek to understand the benefit to scientists could be developed as an ongoing evaluation component from the beginning, possibly using, or informed by, our validated instrument (Additional file 1). The focus of our study was to better understand our two stated research questions: (1) In what ways did working with the teachers on their research project affect the scientists? (2) In what ways did the research experience affect scientists’ practice? Other questions, of particular interest to similar studies in the future, might also be useful to better understand the value of these kinds of partnerships.

Overall, here we show that scientists potentially perceive great benefits from these kinds of partnerships. While the primary intent and scope of this study was to document the benefits accrued to the scientists in RET-like projects, our results and recommendations described above may also have broader relevance to other kinds of scientist-teacher partnerships.

## Supplementary Information

**Additional file 1. **PCPPireTeach and GABI RET Mentor [Scientist] Survey. Validated survey used for the scientists described in this study.

## Data Availability

The data used to prepare this report are contained in the summative evaluation document referenced as Davey (2017) below. The validated scientist survey is appended as an Additional file 1.

## Abbreviations

GABI: Great American Biotic Interchange
K-12: Grades kindergarten through 12 in the U. S. public school system
mya: Million years ago
NSF: U. S. National Science Foundation
PD: Professional development
RET: Research Experiences for Teachers (NSF)
REU: Research Experiences for Undergraduates (NSF)
STEM: Science, Technology, Engineering, Technology
