# Supplementary material for: Scientists benefit greatly from K-12 partnerships: the Panama Research Experiences for Teachers project
Source: Evolution (N Y). 2022 Dec 19;15(1):20. doi: 10.1186/s12052-022-00177-z (PMC9762642; doi:10.1186/s12052-022-00177-z)
Supplement: Supplementary file 1 — Additional file 1. PCPPireTeach and GABI RET Mentor [Scientist] Survey. Validated survey used for the scientists described in this study. [file 12052_2022_177_MOESM1_ESM.docx]

**Additional file 1. PCPPireTeach and GABI RET Mentor [Scientist] Survey**

*This survey seeks to help us better understand your role and experiences with teachers while you participated in the Panama Research for Teachers project. Five cohorts were included in this program from 2012 to 2016 with cohorts 1 to 2 participating in PCPPireTeach and cohorts 3 to 5 were participating in GABI RET. Please consider your experiences when you were in Panama with the teachers, as well as other activities that may have occurred. We use the terms “mentor” and “mentoring” below to indicate the work that you did, your collaborations with the teachers during this Panama experience, and any phases of professional development. Please complete all items as thoroughly as possible.*

1. During which cohort(s) did you participate?

2012 (Cohort 1)

2013 (Cohort 2)

2014 (Cohort 3)

2015 (Cohort 4)

2016 (Cohort 5)

2. Did your understanding of teaching and learning change as a result of working with your teacher partners? If so, how?

3. If you had an opportunity to visit a classroom, how did your visit impact you? If you visited more than once, how did your visits change over time?

4. Explain how working with teachers on the Panama project influenced your implementation of the scientific process in your own work.

5. What did you learn about mentoring from working with the teachers during this experience? What surprised you most about these interactions?

6. Please rate the following on your level of agreement BEFORE and AFTER your Panama experience.

Please use the following scale:

5 = Strongly Agree

4 = Agree

3 = Neither Agree nor Disagree

2 = Disagree

1 = Strongly Disagree

|  | Rating  BEFORE | Rating AFTER |
| --- | --- | --- |
| Working with and mentoring teachers benefits scientists as science professionals (in terms of career and identity as a professional scientist). |  |  |
| Working with and mentoring teachers benefits scientists as educators (in terms of attitudes toward teaching and learning as educators). |  |  |
| Working with and mentoring teachers benefits scientists as individuals (in terms of fulfillment and giving back to the community). |  |  |
| It is a benefit to teachers to have a scientist as a mentor. |  |  |
| I am able to communicate science concepts simply and effectively (in layman’s terms). |  |  |

7. Please indicate the number of each type of continued collaboration you have had with any of the teachers.

|  | Number |
| --- | --- |
| Developed lesson plans |  |
| Classroom visits |  |
| Conference presentations (papers and/or posters) |  |
| Co-authored a research paper |  |
| Virtual lecture |  |
| Offered field experiences |  |
| Other, please describe. |  |

8. To what extent do you agree with each of the following statements about your experience as a mentor?

Please use the following scale:

5 = Strongly Agree

4 = Agree

3 = Neither Agree nor Disagree

2 = Disagree

1 = Strongly Disagree

|  | Rating |
| --- | --- |
| Working with and mentoring teachers as part of this research experience enabled me to see how they benefited from our interactions. |  |
| I benefited from working with and mentoring teachers as part of this research experience. |  |
| My experiences in working with and mentoring teachers as part of the Panama project were positive and I would participate again in the future. |  |
| Working with and mentoring teachers changed my views about the demands and needs of K-12 teachers. |  |
| I have better understanding of working in the classroom with K-12 students. |  |
| As a result of my participation, I have a greater interest in teaching in K-12 settings. |  |
| I would like to work with and mentor (a) K-12 teacher(s) in the future. |  |
| As a result of mentoring teachers, I gained a better understanding of how to work with students. |  |
| As a result of mentoring teachers, I gained better communication skills. |  |
| I feel it is important for teaches to have field-experiences as part of their educational training. |  |
| I feel it is important for students to have inquiry-based learning opportunities. |  |

9. Has working with teachers as a mentor made you a better scientist? If so, in what ways?

10. What changes, if any, have you made in your work as a scientist as a result of mentoring teaches as part of the Panama experience?

11. Do you have any additional comments to add?
